# Quantum Dot Fluorescent Imaging: Using Atomic Structure Correlation Studies to Improve Photophysical Properties

**DOI:** 10.1021/acs.jpcc.3c07367

**Published:** 2024-01-31

**Authors:** Ruben Torres, Lucas B. Thal, James R. McBride, Bruce E. Cohen, Sandra J. Rosenthal

**Affiliations:** † Department of Chemistry, Vanderbilt University, Nashville, Tennessee 37240, United States; ‡ Vanderbilt Institute of Chemical Biology, Vanderbilt University, Nashville, Tennessee 37240, United States; § Vanderbilt Institute for Nanoscale Science and Engineering, Vanderbilt University, Nashville, Tennessee 37240, United States; ∥ Department of Electrical and Computer Engineering, Vanderbilt University, Nashville, Tennessee 37240, United States; ⊥ The Molecular Foundry and Division of Molecular Biophysics & Integrated Bioimaging, Lawrence Berkeley National Laboratory, Berkeley, California 94720, United States; # Department of Pharmacology, Vanderbilt University, Nashville, Tennessee 37240, United States; 7 Department of Chemical and Biomolecular Engineering, Vanderbilt University, Nashville, Tennessee 37240, United States; 8 Vanderbilt Interdisciplinary Materials Science Program, Vanderbilt University, Nashville, Tennessee 37240, United States

## Abstract

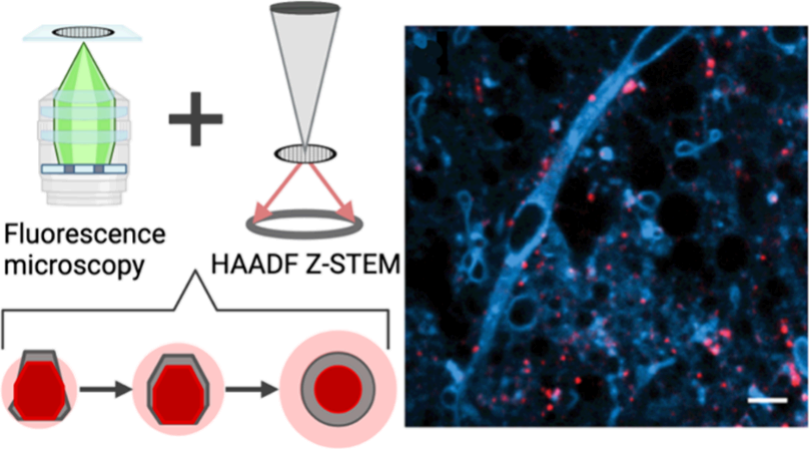

Efforts to study intricate, higher-order cellular functions have called for fluorescence imaging under physiologically relevant conditions such as tissue systems in simulated native buffers. This endeavor has presented novel challenges for fluorescent probes initially designed for use in simple buffers and monolayer cell culture. Among current fluorescent probes, semiconductor nanocrystals, or quantum dots (QDs), offer superior photophysical properties that are the products of their nanoscale architectures and chemical formulations. While their high brightness and photostability are ideal for these biological environments, even state of the art QDs can struggle under certain physiological conditions. A recent method correlating electron microscopy ultrastructure with single-QD fluorescence has begun to highlight subtle structural defects in QDs once believed to have no significant impact on photoluminescence (PL). Specific defects, such as exposed core facets, have been shown to quench QD PL in physiologically accurate conditions. For QD-based imaging in complex cellular systems to be fully realized, mechanistic insight and structural optimization of size and PL should be established. Insight from single QD resolution atomic structure and photophysical correlative studies provides a direct course to synthetically tune QDs to match these challenging environments.

## Introduction

Imaging fluorescent probes to study fundamental life science processes has been a keystone technique for over a century.^1−3^ Fluorescent probes fall into three main classes: organic dyes, fluorescent proteins, and inorganic nanoparticles.^3^ These probes may be tethered to biological targets^4−9^ and visualized using optical microscopy. Much work using these classes of probes has been carried out in two-dimensional (2D) samples, consisting of a monolayer of a single cell type in culture. While 2D samples offer a reductionist approach to isolate and observe key processes associated with cellular biochemistry, development, oncogenesis, and communication, they may fail to capture higher order interactions, localizations, and timings. The necessary next step to assess physiological relevance has been to transition to three-dimensional (3D; tissue, organoids) systems under physiologically accurate conditions. However, this step remains challenging due to a lack of bright, low phototoxicity, and photostable probes that can penetrate deep into tissue and enable high frame rate video acquisition. Therefore, ideal fluorescent probes for 3D imaging would be small enough to diffuse into sterically hindered regions while maintaining high photon outputs at longer wavelengths to reduce light scattering within the sample–all while remaining photostable for extended time scales of continuous acquisition.

Fluorescent proteins and organic dyes are widely used probes, each possessing their own set of properties for specific applications.^3,9−12^ Inorganic nanoparticles garnering attention as transformative fluorescent probes for 3D imaging include alloyed upconverting nanoparticles (aUCNP)^13−15^ and single-walled carbon nanotubes (SWCNT).^12^ aUCNPs can produce steep nonlinear first near-infrared (NIR-I) photon emission (photon-avalanching) from second NIR (NIR-II) excitation,^15^ and SWCNTs emit in the NIR-II.^12^ NIR excitation and emission is advantageous for deep imaging,^16^ circumventing cellular autofluorescence and reducing photon scattering (see reviews on upconverting nanoparticles^17,18^). While there are numerous optical probes to choose from, some with new and exciting properties, developing biocompatible probes that are small and photostable and can be imaged through tissue remains an ongoing challenge.^19−21^

Since their initial integration into biological systems,^22,23^ fluorescent semiconductor nanocrystals, often referred to as quantum dots (QDs), have been imaged in 2D samples in direct competition with standard probes such as organic dyes and fluorescent proteins (see reviews on QD fluorescent imaging^24−26^). QDs possess unique photophysical properties that offer promise for 3D samples, including (i) large extinction coefficients and molar absorptivity allowing longer wavelength excitation and emission for high signal-to-background ratios and reduced light scattering in deep-tissue imaging, (ii) resilient photostability for long-term acquisition, and (iii) large two- and three-photon excitation cross sections for multiphoton microscopy.^26−28^ Moreover, typical QD sizes (4–12 nm diameter)^29,30^ can grant access to sterically hindered cellular spaces, as restrictive as neuronal synaptic clefts (15- 20 nm).^31,32^ These properties suggest that QDs have significant potential for imaging biomolecules or complex cellular structures in 3D samples.^33−35^ However, after decades of conventional 2D imaging use, QD-based 3D imaging has yet to fully come to fruition as a standard imaging method. This shortfall is likely a direct result of underperforming PL identified in commercially available QDs, which were designed to have reproducible quantum yields and high brightness in simple buffers and cell systems, but not necessarily long-term chemical and photostability.^36^

Conventional QD development involves iteratively colloidal synthesis followed by measure of static absorption and fluorescence of each batch, with high fluorescence efficiency being the primary benchmark.^37,38^ Traditional ensemble characterization techniques may demonstrate superior QD PL properties; although it is well understood that colloidal syntheses give varying sizes with differing emission maxima that broaden fwhm, the more subtle structural variations are just as important. Detailed electron microscopy (EM) indicates appreciable structural heterogeneity, suggesting the presence of bright, dim, and dark populations within single batches.^36,39^ This discrepancy proceeds undetected in most macroscopic applications, whereas in single-molecule bioimaging, the detrimental effects of structural heterogeneity on PL performance are apparent. Consequently, the large batch-to-batch and interbatch variability with commercial QDs may manifest as unreliable performance which may turn optical microscopists away from QD probes. For QDs to become standard fluorescent probes for 3D samples under physiologically accurate cellular environments, defects in the ultrastructure that attenuate PL performance under these conditions must be identified and corrected, all while maintaining the smallest necessary size. Recent advances in correlating photophysical properties with atomic structure allow for detailed investigation into the atomic-structure-PL-function relationship of individual QDs, guiding synthetic methods to select optimal structural configurations for imaging 3D samples.

## PL Properties Depend on Atomic Structure

QD PL properties are products of their nanoscale and chemical structures. When a semiconductor absorbs a photon, an electron is promoted from the valence band to the conduction band, leaving behind its positively charged hole counterpart. These constituents are Coulombically attracted, and upon recombination, a photon is emitted from the QD. As the QD becomes smaller, the electron and hole are spatially confined with a particular confinement energy. This confinement shifts energy states to higher levels, producing higher energy (blue-shifted) photons, whereas increasing the QD size shifts energy states to lower energy levels (red-shifted). These charge carriers are susceptible to nonradiative recombination by falling into lower energy trap states that reduce their energy or prevent recombination. Epitaxial growth of a wider band gap semiconductor shell onto the QD surface can eliminate dangling bonds.^40^ A wider band gap shell is the optimal approach to permanently passivate the core, because the shell ideally covers all surface atoms. Coordinating ligands may not completely passivate all surface atoms and are dynamic,^41^ allowing chemical species to replace and/or react with surface atoms to promote nonradiative recombination. Another benefit of the core/shell structure is the sheer number of atoms within one particle, each with the capacity to absorb and emit a photon, resulting in their hallmark brightness.

## Organic Shell Affords Biocompatibility and The First Line of Defense

**Figure 1:**
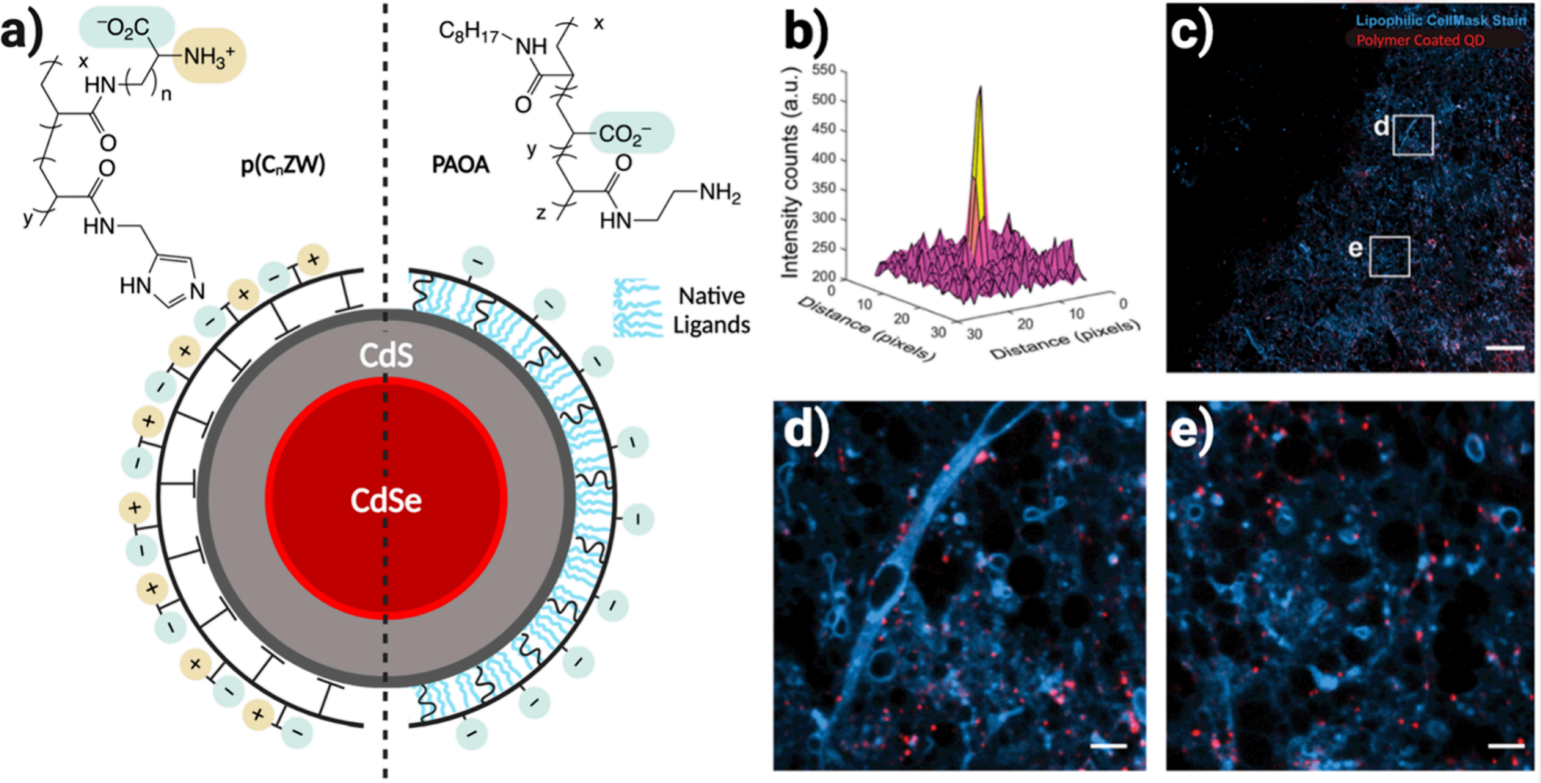
(a) Chemical structures and schematic depictions of coated QD surfaces using a multidentate zwitterionic polymer ligand (left) or an amphiphilic copolymer (right). (b) Surface plot of an amphiphilic copolymer coated QD point spread function imaged 50 μm into a live mouse brain slice. (c) Stitched image of amphiphilic copolymer coated QD conjugates dispersed in a brain tissue (scale bar = 50 μm). (d and e) 10× magnification of various regions captured in the stitched image in (c) (scale bar = 5 μm). Adapted with permission from ref (60). Copyright 2020 Royal Society of Chemistry.

Most QDs are inherently hydrophobic, as they are colloidally synthesized using nonpolar solvents and are capped with nonpolar coordinating ligands. Therefore, an intermediate step using one of the two methods is required to render aqueous QDs. The first method is a ligand exchange whereby the native nonpolar ligands are desorbed from the QD surface and replaced with polar ligands.^42,43^ We highlight a recent and promising zwitterionic polymer coating (Figure 1a, left) that contains a mixture of outward-facing positively and negatively charged functional groups to solubilize and yield a net-neutral surface.^44^ This method is common but consequently risks PL performance as the native ligands serve a vital role in passivating surface trap states, therefore swapping with different ligands can negatively affect PL properties.^45−50^ Additionally, the hydrophilic ligands can desorb from the surface or be exchanged by thiols or amines found in cells and biological media (see reviews on water-soluble QD coatings^45,51^). The second method is a surface coating around the native QD ligands by using an amphiphilic polymer (Figure 1a, right). The nonpolar groups intercalate into the native ligand network, and the hydrophilic groups face outward to solubilize the entire QD (Figure 1a, right). Amphiphilic polymers preserve PL properties since native ligands are undisturbed and the polymer adds additional protection to the QD surface, which typically yields brighter QDs,^45^ although this approach adds ultimately to its hydrodynamic diameter.^52−54^ Biocompatible QDs may then be functionalized with small molecule affinity handles or antibodies to label specific biomolecules.^55^Figure 1b–e shows amphiphilic polymer coated QDs imaged in striatal mouse brain slices.

## Correlation of Photophysics with Atomic Structure Identifies Specific Defects

Conventional PL sampling techniques may mask decreased PL from defective QDs and treat all QDs as structurally identical.^36^ PL properties are strictly dependent on explicit atomic structure where minor structural variations such as addition of a single atomic layer of core or shell material drastically influences charge carrier dynamics, thus affecting PL performance.^56^ Now microscopists are predominantly concerned with observing detailed spatiotemporal dynamics of single biomolecules—by extension the PL of single fluorescent probes—thus, photophysical properties of single QDs must be investigated. A lack of insight into how specific atomic arrangements influence single nanoparticle PL is a key reason keeping QDs from becoming standard fluorescent probes for imaging 3D samples. Therefore, detailed correlative characterization between atomic structure and photophysics on individual QDs will provide a means to further improve QD efficacy for 3D imaging.

**Figure 2:**
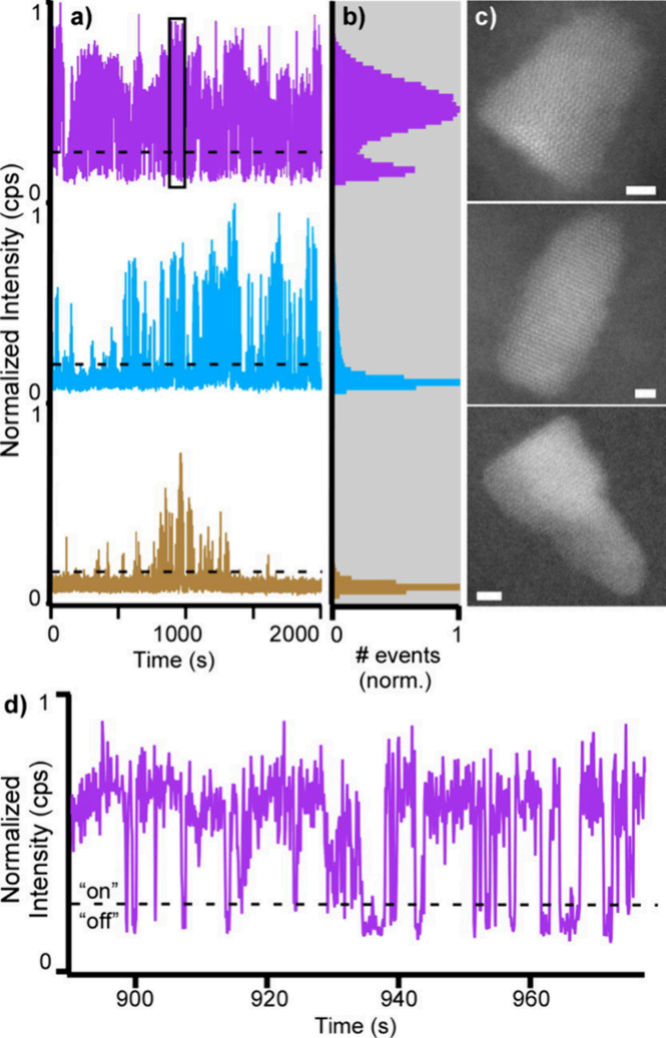
Correlated fluorescence and structural data for individual QDs. (a) Full fluorescence intensity transients with (b) corresponding photoluminescence intermittency histograms for the 3 QDs whose structures are shown in (c). (d) The boxed truncated portion of the topmost fluorescence intensity transient in (a) shows the “on” and “off” states of a fluorescent QD. Scale bars in (c) are 2 nm. Reprinted with permission from ref (36). Copyright 2014 American Chemical Society.

The first application of a correlative technique used to investigate commercially available QD655s (CdSe/CdS; Life Technologies, QDOT 655) commonly used in biological imaging is shown in Figure 2. Since commercial QDs possess high quality ensemble PL spectra, the influence from subtle structural variations on PL can be understood. Here, polystyrene latex spheres are deposited onto a SiO_2_ transmission electron microscopy (TEM) support to act as fiducial markers so that fluorescence (Figure 2b) and structural (Figure 2c) data can be reliably associated with the same QD655. A SiO_2_ support is crucial to the success of this correlative technique as standard carbon supports quench QD PL, and SiN supports generate high levels of background fluorescence. High annular dark field scanning transmission (HAADF-STEM) provides high contrast to locate particles more easily, while also minimizing sample damage caused by the electron beam. As shown in Figure 2, structurally and morphologically similar QD655s viewed by HAADF-STEM have drastically different PL intermittency (blinking) profiles between them, highlighting the “masking effect” that ensemble PL spectra can have.

**Figure 3:**
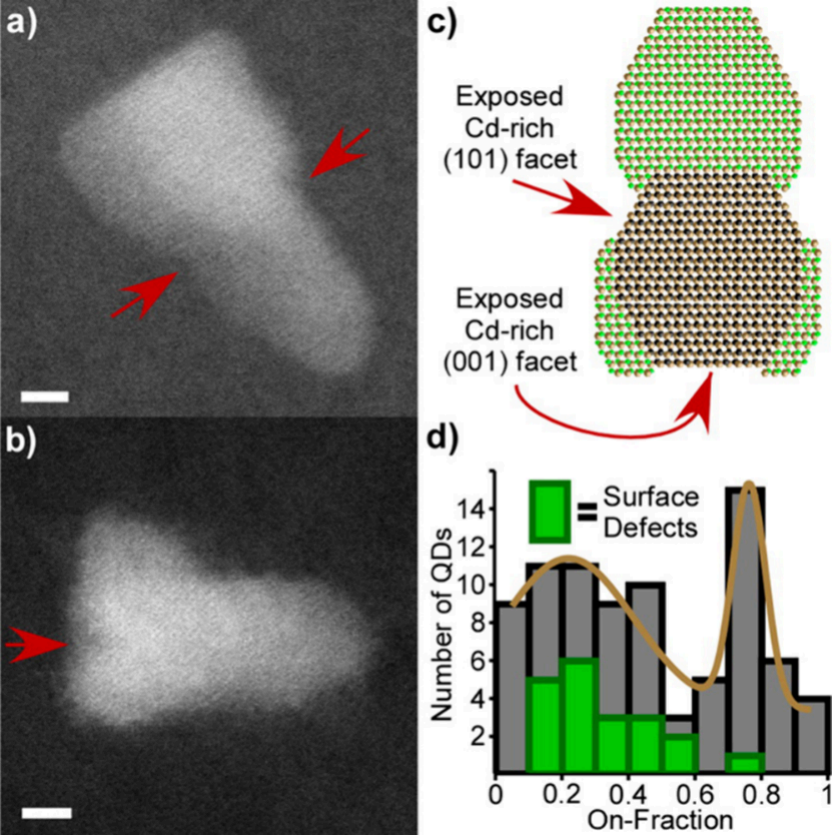
Characteristic defects for QDs with a low on-fraction. (a) QD exhibiting an exposed core Cd-rich facet at (101) and (b) (001). (c) Representative QD structure indicating Cd-rich facets onto which lack of shell growth results in a lower on-fraction. (d) Distribution of all observed QDs as a function of on-fraction with QDs containing surface defects (a) and (b) overlaid. Scale bars are 2 nm. Reprinted with permission from ref (36). Copyright 2014 American Chemical Society.

**Figure 4:**
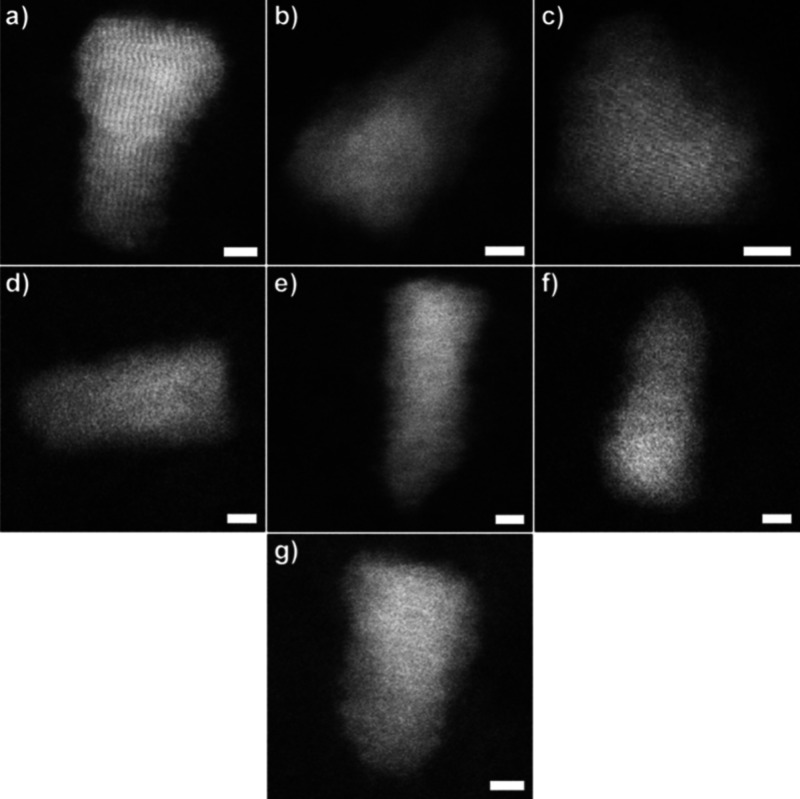
Structures of permanently nonradiative, or “dark”, CdSe/CdS core/shell QDs. Structural defects such as stacking fault in core region (a), asymmetrical shell growth (b), little or no shell material (c). (d–f) “Dark” QDs that do not show obvious surface or internal defects. Scale bars are 2 nm. Reprinted with permission from ref (36). Copyright 2014 American Chemical Society.

Since epitaxial growth of a wider band gap semiconductor shell (CdS) onto the core eliminates lower energy trap states that would attenuate PL performance,^40^ the impact of specific structural defects of shell growth on individual QD PL can be studied as well. In Figure 3a,b, CdS epitaxy is preferred on Se-rich facets,^57^ resulting in a population of QD655s with exposed cores at Cd-rich facets at (101) and (001). Populations of QD655s containing exposed core facets spend less time in emissive “on” states (Figure 3c). Exposed Cd-rich facets are likely capped by ligands that can eliminate low energy trap states but due to steric bulk these ligands cannot completely passivate all surface atoms, leaving exposed atoms to oxidative degradation.^58^ Unlike conventional single particle fluorescence techniques that only identify strongly fluorescence particles, this method uniquely has the capability to identify nonemissive “dark” QDs that would otherwise remain undetected (Figure 4). Of the QD655s surveyed, ∼8% were observed in the “dark” state, inferring a remarkable amount of unobservable QDs that still partake in biomolecule labeling. Of more concern, 31% of particles exhibited high “on-fraction” or periods of time where the particle emitted. It is likely that only this subset of particles is observable, leaving roughly 70% of all functionalized probes undetectable during a single fluorescence experiment.

## Commercial QDs Are Not Compatible for 3D Samples

The quality of data and subsequent confidence in interpretation strictly depend on the PL quality of the fluorescent probe used for biological imaging. As stated earlier, fluorescent probes are often engineered for and evaluated in 2D samples, while transitioning into 3D samples introduces unforeseen obstacles that affect PL performance. QD-based 2D imaging is conducted under “mild” imaging conditions, such as general biological buffers with low oxidative species and lower excitation fluences. Under these conditions, photooxidative degradation at exposed Cd core facets happens at rates slow enough that degradation outgains data acquisition. In 3D samples within physiologically accurate buffers, significantly higher concentrations of oxidative species, such as oxygen, exist, and higher excitation fluences are needed, thus creating harsh photooxidative environments for not only QDs, but organic dyes and fluorescent proteins. It is in this environment that the structural heterogeneity revealed by the correlation study begins to grossly affect the QDs performance.

**Figure 5:**
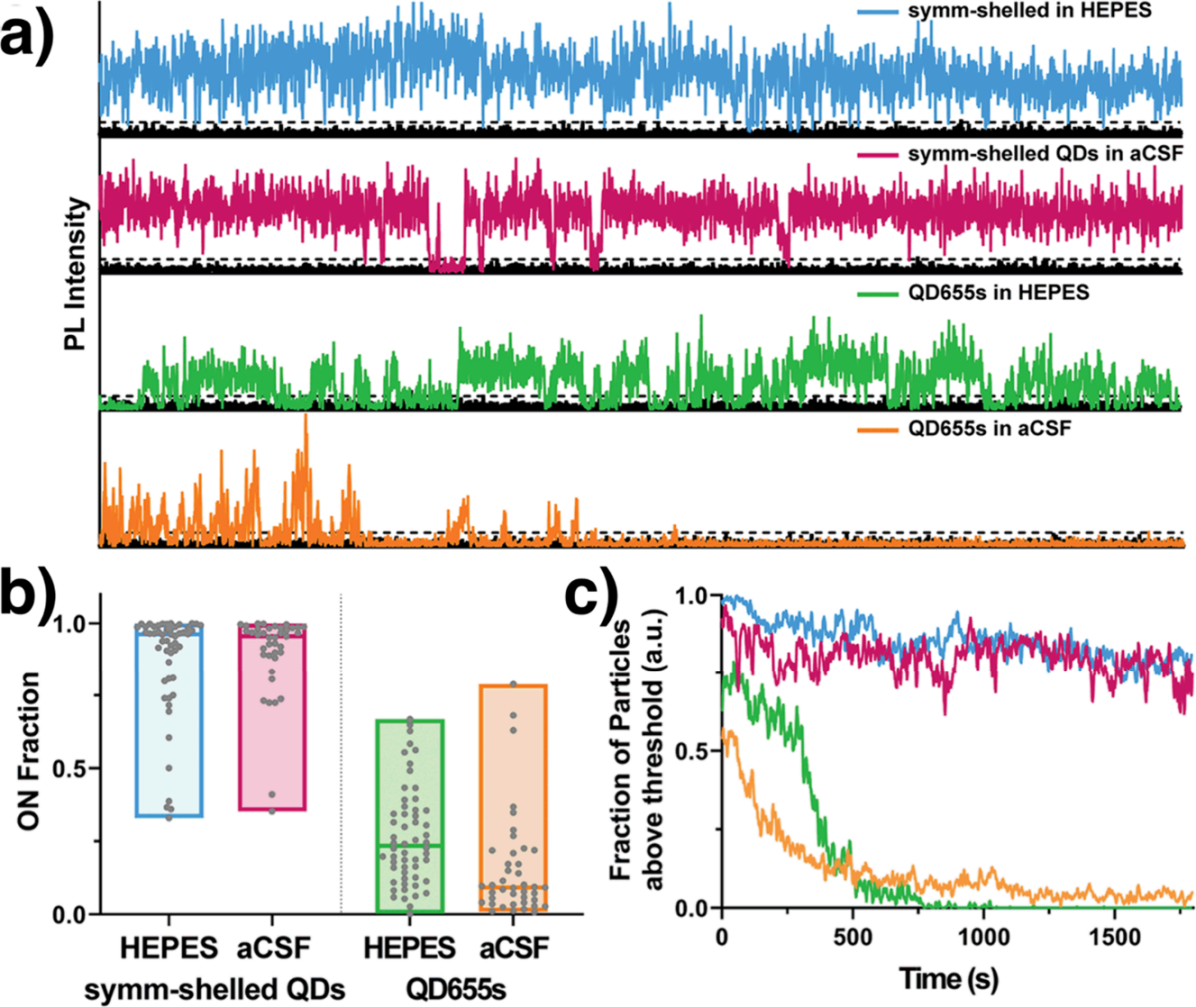
Time series and blinking behavior of single QDs. (a) Representative fluorescence intensity transients for symm-shelled QDs and QD655s in both HEPES and oxygenated aCSF. (b) Comparison of ON fraction populations under each condition (*N* ≥ 40 QDs). (c) Comparison of photobleaching profiles for symm-shelled QDs vs QD655s under each condition (*N* > 40 QDs. Reprinted with permission from ref (60). Copyright 2020 Royal Society of Chemistry.

In 2013, Chen and co-workers synthesized a compact, thick CdS shelled QD with both high quantum yield and improved stability.^59^ This was further investigated in the context of PL performance in a physiologically accurate buffer rich in oxygen, such as artificial cerebrospinal fluid (aCSF, 95% O_2_) and was shown that uniform CdS shell symmetry protects the core from photooxidative degradation.^60^ These symmetrically shelled QDs (symm-shelled QD) have a similar amount of shell, but the commercial QD655 shells are localized mostly on one facet. Single QD PL analysis further demonstrates the deleterious effects that uneven shell coverage has on the QD655 PL when dispersed in aCSF. Figure 5 shows high blinking suppression on fractions in symm-shelled QDs. QD655s experience lower blinking suppression and smaller on fractions in a control buffer (HEPES) and the lowest blinking suppression and smallest on fraction in aCSF. To simulate times scales of a long-term study, both QDs were excited continuously for 30 min. Strikingly, nearly all QD655s photobleach within minutes, and >80% of symm-shelled QDs remain luminescent over the entire excitation duration (Figure 5c), emphasizing the importance of symmetrical shell coverage over the core. While the exact role that oxygen has in QD PL performance remains unclear, there are several QD-related factors that may influence how oxygen behaves: (i) the structural quality,^61−68^ (ii) the medium, in air^64,67^ or pure oxygen,^62,63,66^ and (iii) the phase, in solution^69,70^ or thin film.^61,64,66^ In the context of this study, it is likely that oxygen in aCSF photocorrodes QD655s at exposed core facets and degrades coordinating ligands, leading to irreversible photobleaching, as evidenced by plain CdSe core QDs undergoing irreversible photocorrosion in oxygen,^71^ while “giant-shell” CdSe/CdS QD experience suppressed blinking and persistent photostability.^72^ In the field of neuroscience commercial QDs have been dynamically imaged in 3D systems like acute brain slices^34,35,73^ and organotypic slices^74^ but may have faced difficulties resulting from shell deficiencies. Defective QDs cannot survive harsh 3D imaging conditions; therefore, QDs with optimized structural arrangements for maximum efficacy are of the utmost importance, with the aspiration of becoming accessible to biology-related disciplines.

## Prospects for the Future

**Figure 6:**
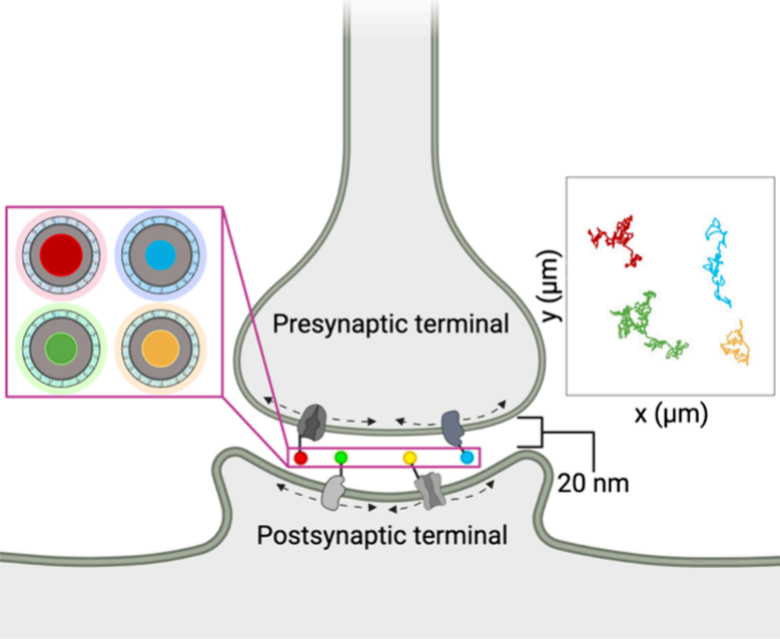
Hypothetical schematic of amphiphilic copolymer coated QDs of four different emission wavelengths with hydrodynamic diameters small enough to access a synaptic cleft. Each QD is functionalized to specifically label four different arbitrary membrane proteins and then tracked using optical microscopy. Reconstructed hypothetical trajectories of QDs corresponding to dynamic movement of membrane proteins are plotted on the right.

Sterically hindered cellular spaces, such as brain tissue, require the smallest fluorescent nanoparticle possible. For instance, the active communication zone (synaptic cleft) between neurons carries out intricate electrochemical signaling that is poorly understood. QDs have the capability to access the synaptic cleft (15–20 nm across) and investigate the spatiotemporal processes governing neuronal signaling. The functionalized symm-shelled QDs mentioned previously have a hydrodynamic diameter of 15 nm^54^ that allows access to synaptic clefts. “Ultrasmall” (2 nm diameter) CdSe QDs^75,76^ can be shelled to produce <10 nm CdSe/CdS QDs, increasing accessibility to more narrow synaptic clefts, at a slight loss of relative brightness. Neuronal protein-specific affinity handles can be conjugated to biocompatible QDs of different emission wavelengths, allowing for multiplexed labeling. Since QDs exhibit narrow Gaussian emission spectra,^25^ multiplexed imaging can be more easily implemented than organic dyes or fluorescent proteins^77^ to yield high resolution spatiotemporal data^78^ for several principal proteins during the entire signaling event, as depicted in Figure 6. The “ideal” QD possesses the ability to reach sterically hindered spaces while maintaining high PL intensity; however, atomic structure photophysical correlation is needed to develop the “ideal” QD.

An emergent role for QD biological imaging is in correlative light and electron microscopy (CLEM).^79^ CLEM employs both optical and electron microscopy on the same biological sample. Dynamic fluorescence data are initially collected on a live biological sample (2D or 3D). Then the sample is cryogenically frozen, and the ultrastructure is collected using electron microscopy. QD probes would serve as a “one-step” label where specific proteins of interest can be dynamically imaged using QD fluorescence, and then those same QDs, identifiable to TEM, can serve as general registration fiducials^80^ or target-specific fiducial markers to collect ultrastructure of the protein of interest.

## Conclusion

Fluorescent imaging carried out in simple cell cultures has created immense insight into biology and medicine, although as more complex questions have arisen, more physiologically relevant systems have been developed. This concerted effort to image 3D samples under physiologically accurate conditions demands better fluorescent probes that are designed for these conditions. While no fluorescent probe is ideal, QDs have demonstrated potential in terms of size and stability. Initial implementation in 3D sample imaging has identified a negative QD PL performance caused by fine structural defects. Additional structural photophysical correlation studies are needed so that core/shell structure can be optimized for imaging 3D samples. Evidently, a shell thick enough to surround all core facets is critical to preserve QD PL in 3D samples; nonetheless, a balance must be established between shell coverage and overall probe size: if shell coverage is too thick, access to sterically hindered regions will be blocked, but if shell coverage is too thin, PL plummets. Additionally, a systematic study into other molecular species across several physiologically accurate buffers that drive QD photobleaching can lead to improved polymer coatings to suppress these effects. Insight gained could guide synthetic methods to improve monodispersity or lead to novel QD purification techniques for selecting specific structural arrangements. Guidance from atomic structure photophysical correlation studies will lead to optimized QD probe design, where long-term studies in 3D samples will uncover novel mechanisms of living systems.
